# The effect of pre-treatment and anaerobic digestion for pathogens reduction in agricultural utilization of sewage sludge

**DOI:** 10.1007/s11356-022-23164-9

**Published:** 2022-09-23

**Authors:** Alicja Machnicka, Klaudiusz Grübel

**Affiliations:** Faculty of Materials, Civil and Environmental Engineering, Departure of Environmental Protection and Engineering, University of Bielsko-Biala, Willowa 2 Str, 43-309 Bielsko-Biala, Poland

**Keywords:** Waste-activated sludge, Disintegration, Anaerobic digestion, Hygienization, Biogas

## Abstract

The aim of the research work was to explain the possibilities of application of waste activated sludge (WAS) pretreatment processes prior to anaerobic digestion (mesophilic fermentation). Hydrodynamic disintegration and freezing/thawing disintegration methods were used. Based on the microbiological and parasitological analyses, a significant decrease in pathogenic bacteria, coliphages, and parasite eggs was observed. The number of bacteria analyzed (*Salmonella* sp., *Escherichia coli*, *Clostridium perfringens*) and coliphages were reduced from 19.3to 42.3% after hydrodynamic cavitation. A similar effect was achieved for destruction by freezing/thawing with dry ice between 7.8 and 14.9%. The effectiveness of parasite eggs reduction (*Ascaris* sp., *Trichuris* sp., *Toxocara* sp.) for these disintegration methods ranged from 10.7 to 29.3%. The highest results were observed for the hybrid disintegration method (hydrodynamic cavitation + dry ice disintegration) caused by a synergistic effect. *Salmonella* sp. in 1 g_d.w._ decrease about 69.7%, *E. coli* by 70.0%, *Clostridium perfringens* by 38.4%, and coliphages by 48.2%. Disruption of WAS by a hybrid method led to a reduction in the number of helminth eggs *Ascaris* sp. (63.8%), *Trichuris* sp. (64.3%), and *Toxocara* sp. (66.4%). After anaerobic digestion under mesophilic conditions, an additional reduction of analyzed bacterial pathogens and helminth eggs were observed. The introduction of hybrid disintegrated WAS to the fermentation chamber resulted in higher efficiency in decrease (from 1 to 23%) in comparison to the control sample (70%WAS + 30%DS (inoculum-digested sludge)).

## Introduction

Sewage sludge comes into existence in wastewater treatment plants as a peculiar waste of wastewater treatment processes. The legal regulations regarding sewage sludge management have restrictions on the presence of volatile substances, disease-causing pathogenic organisms (viruses, bacteria, protozoa, and parasite eggs), heavy metals, and inorganic ions, along with dangerous chemicals from industrial wastes, household chemicals, and pesticides (López et al. 2019; Li et al. 2021).

However, sewage sludge has intrinsic fertilizer values. Sludges from the biological process of wastewater treatment are abundant in organic substances, nitrogen, phosphorus, calcium, magnesium, sulfur, and microelements essential to the life of plants and the soil fauna. Exploiting the fertilizer value of sewage deposits has great importance in the case of environmental protection, which is a serious problem. Sewage sludge is increasingly used for fertilizing agricultural lands and forests and for the recultivation of devastated areas. However, a very important technical problem is always presented from the point of view of large hydration, mass and sanitary danger. In addition to the content of heavy metals and toxic substances and microbiological contaminations, the usefulness of sewage deposits for use in farming constitutes the basic criterion. Bacteria, viruses, and parasites are the most commonly transmitted fecal–oral pathogens. These microorganisms are transmitted in the sludge in which they accumulate (Gopi Kumar et al. 2012; Lakshmi et al. 2014; Lopes et al. 2017; Jiang et al. 2020).

With regard to pathogens, the requirements are based on the principle of reducing and eliminating pathogens from sewage sludge destined for agricultural use. In the technological process of wastewater treatment plants, elimination of microorganisms is achieved by appropriate treatment (conditioning) of the sludge, e.g., in thermal processes, which ensures the reduction of the amount of pathogens and the appropriate microbiological quality of the sludge (Hu et al. 2011; Lloret et al. 2012; Zhou et al. 2015; Eng et al. 2015; Wójcik et al. 2017; Luukkonen et al. 2020). The sanitary index describes the content of pathogenic bacteria and eggs of intestinal parasites in the sludge on the basis of indirect conclusions. Not so long ago in Polish law, the species that was a sanitary indicator among bacteria was lactose-positive *Escherichia coli*. Currently, according to the ordinance of the Minister of the Environment (2015), the presence of the lactose-negative bacteria *Salmonella* sp. is used as a new indicator.

Sewage sludge is often polluted by many types of helminths. The source of parasite eggs in sewage sludge is human and animal feces, which can also cause soil or food contamination. The parasite indicators *Ascaris* sp., *Trichuris* sp., and *Toxocara* sp. are being used in the sanitary estimation of sewage sludge (Pike et al. 1988; Jiang et al. 2020).

The risk of contamination of the environment with pathogenic microorganisms from sewage sludge causes the constant search for methods of their destruction. Sanitary safe sludge can be used for agricultural purposes. Disintegration of activated sludge and fermentation are the most applied processes for the stabilization and hygienization of sewage sludge. The disintegration of sewage sludge can occur by mechanical, chemical, and biological methods and reduces the flock size, disrupts the cell walls, and releases organic material from the cells to the liquid phase (Doğan and Sanin 2009; Liao et al. 2016; Machnicka et al. 2017; Grübel et al. 2018; Khanh Nguyen et al. 2021). The next step in sludge technology is anaerobic digestion conducted under psychrophilic, mesophilic, thermophilic, and two-step processes. In recent years, most wastewater treatment plants have realized this stabilization under mesophilic conditions. The main benefits of fermentation of sewage sludge are its stabilization and reduction of sludge mass, methane production, hygienization, and improvement of the drainage properties of the digested sludge (Zhen et al. 2017; Liu et al. 2019; Khanh Nguyen et al. 2021; Hoang et al. 2022).

Therefore, removal of pathogens by pretreatment of waste activated sludge (WAS) and anaerobic digestion allows applying sludge as a fertilizer. This paper presents results from the hygienization of WAS by pretreatment and anaerobic digestion (mesophilic fermentation).

## Materials and methods

Waste-activated sludge (WAS) was collected (from secondary settling tanks) from a wastewater treatment plant (WWTP) in the Silesian region of Poland, working in according to the Enhanced Biological Nutrient Removal (EBNR) processes.

The test material in two stages was collected (first stage—for hygienization by hydrodynamic cavitation and freezing/thawing process, second stage—for hygienization by anaerobic digestion).

### Pre-treatment of WAS

#### Hydrodynamic disintegration

Hydrodynamic disintegration of WAS was done with the use of a pressure pump (12 bar) equipped with a constructed cavitation nozzle (Grübel and Suschka 2015; Machnicka et al. 2015). Pumping 25 l of WAS through the nozzle took 3 min. The process was underway for 30 min, which corresponded to six multiplicity flow by cavitation nozzle.

#### Disintegration by freezing/thawing

For destruction of WAS, the dry ice was used in the volume ratios of the WAS to dry ice − 1:1. In our previous work, we dealt with the efficiency of sludge disintegration and then anaerobic digestion supported with the use of dry ice (Nowicka et al. 2014; Nowicka and Machnicka 2015).

#### Disintegration by hybrid methods (WASDH)

**Fig. 1:**
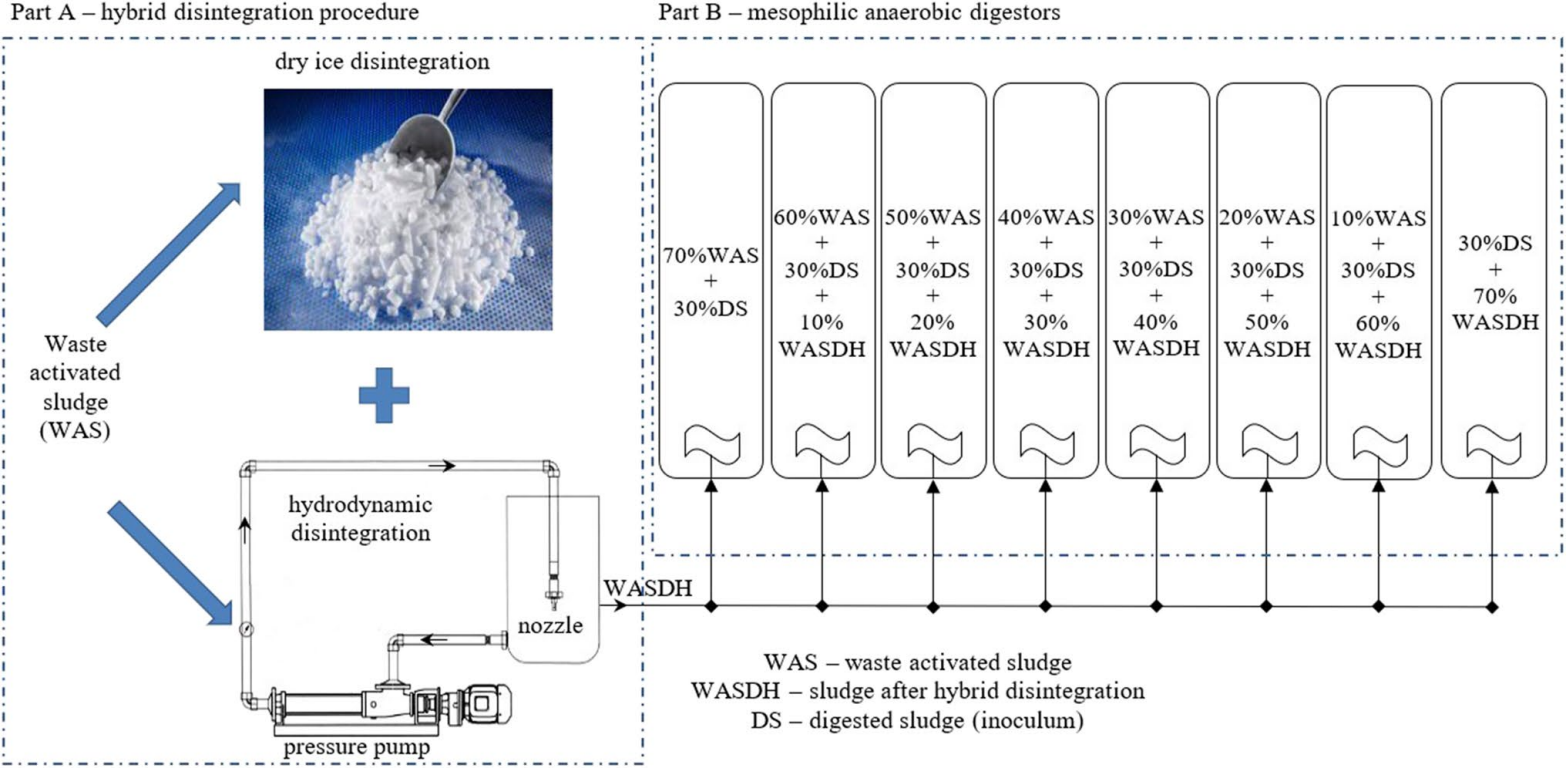
Experimental setup: Part A – dry ice and hydrodynamic disintegration installation; Part B – mesophilic anaerobic digestion

Samples after 30 min of hydrodynamic disintegration and dry ice freezing/thawing process was named as hybrid method (Fig. 1).

#### The fermentation experiments

The anaerobic digestion experiments were performed in laboratory scale using fermenter chambers (2.5 L). The processes were taking place in thermostatic chamber, with constant temperatures 35 ± 1 °C by 25 days.

The fermentation was performed as follows: fermenter 1 were fed with WAS (70% vol.) and inoculum (DS – digested sludge; 30% vol.). Fermenters 2–8 were fed with (% vol.): 10%WASDH; 20%WASDH, 30%WASDH, 40%WASDH, 50%WASDH, 60%WASDH, and 70%WASDH (Fig. 1).

The dosage of inoculum has been optimized in previous studies in terms of minimizing the lag-phase and decreasing the disturbance of the fermentation process.

Liquid displacement method was used for the daily measurement of produced biogas. Methane concentration in the biogas was measured using GFM416 analyzer (OMC Envag, Poland).

The investigations presented here were repeated 10 times and (mean values, *n* = 10) arithmetic average and standard deviation were carried out. The standard deviation was determined according to the estimator of the highest credibility in STATISTICA 6.0.

### Microbiological analysis

#### Bacteria and coliphages

The studies related also to the total number of bacteria contained in the WAS before and after the destruction (disintegration by hydrodynamic cavitation and dry ice) and anaerobic digestion (mesophilic fermentation).

Detection and enumeration of *Salmonella* sp. was according to Holt et al. (1994) with using specific culture media. In order to verify the taxonomic classification of Salmonella sp., the API 20E biochemical test was applied. Detection and enumeration of *E. coli* and *Clostridium perfringens* were investigate according to methodology presented in (Grübel and Suschka 2015).

Detection and enumeration of bacteriophages was investigate according to ISO 10705–2:^2000^.

#### Helminth parasites

The studies related also to the total number of parasites eggs contained in the WAS before and after each methods of disintegration and fermentation process. In Poland, the parasitological indicators belonging to the evaluation of the sanitary state of soil (i.e., after introduction of WAS) are live eggs of parasites belonging to nematodes of the genera *Ascaris* spp., *Trichuris* spp. and *Toxocara* spp. The methods of their detection and determination in samples have been developed and contained in the Polish Standard No. PN-Z19000-4:^2001^.

Eggs of *Toxocara* sp. were being isolated from investigated sample by shaking, centrifugation, filtration of flotation suspension processes, and microscopic observation (Zdybel et al. 2019).

The microscope was used in parasitological studies—Nikon Eclipse TS100 coupled with camera MoticamPro 252A (Conbest, Poland) allowed also for size measurements by a program Images Advanced 3.2. Additionally, counter Scan 500 (Interscience Microbiology, France) were used for counts colonies on Petri dishes.

## Results and discussion

The evaluation of a sanitary WAS is based on the inference of the presence of pathogenic bacteria and eggs of intestinal parasites. Its purpose is to detect the so-called sanitary indicator. National and international regulations (European Commission 2000; Council Directive 86/278/EEC; US EPA 2003) dictate that sludge should be stabilized and hygienized before land introduction.

Sewage sludge is characterized by a large number and diversity of microorganisms, including pathogens. The recommended indicators for sanitary assessment of sewage sludge are *Salmonella* sp., *Escherichia coli*, fecal coliforms, enteric viruses, sulfate-reducing *Clostridia*, and parasite eggs. Therefore, it is necessary to look for methods enabling their efficient reduction or total elimination. As a consequence, these techniques allow the possible use of sludge in agriculture. Therefore, the knowledge of selected hygienic indicators in sewage sludge permits making decisions about its use.

**Fig. 2:**
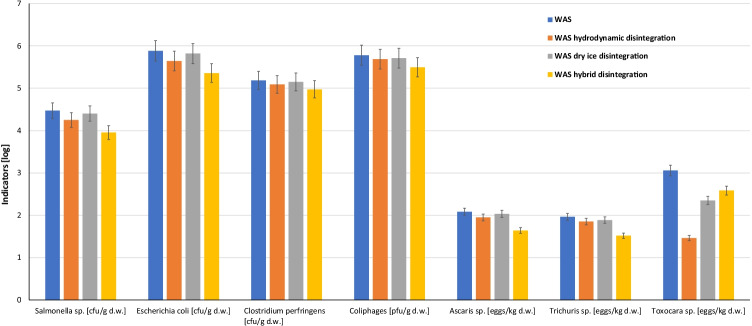
The impact of WAS disintegration on bacterial number and helminth indicators

**Fig. 3:**
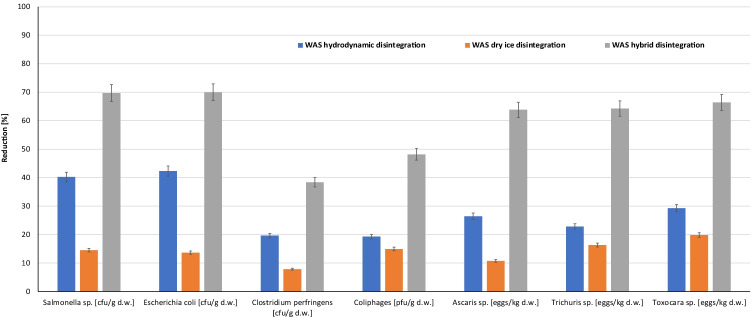
The impact of WAS disintegration on reduction of bacterial number and parasites eggs

The removal of pathogenic bacteria and helminths from waste activated sludge by hydrodynamic destruction and freezing/thawing can help considerably in the reduction of the transfer operation of illnesses. The mechanical process and freezing/thawing can cause the destruction of microorganisms and thus contribute to a partial hygienization of WAS, which was confirmed by the results of the microbiological and parasitological analyses (Figs. 2 and 3).

After hygienization by hydrodynamic cavitation and dry ice, a gradual decrease in the population count of bacteria was observed (Fig. 2). According to Polish law, *Salmonella* sp. removal is imperative for the agricultural use of sewage sludge (Minister of the Environment 2015). After 30 min of hydrodynamic cavitation of sludge, microorganism disintegration resulted in the elimination of *Salmonella* sp. from 4.47 log to 4.24 log cfu/g_d.w._ The number of *Salmonella* sp. in 1 g_d.w._ (the volume ratio of WAS to dry ice was 1:1; freezing/thawing) was reduced by approximately 14.5% (Fig. 3).

Depending on disintegration by hydrodynamic destruction and freezing/thawing, a reduction number of *E. coli* in WAS of approximately 42.3% (from 5.88 log to 5.64 log cfu/g_d.w._) and 13.7% (5.88 log to 5.82 log cfu/g_d.w._) was observed, respectively (Figs. 2 and 3).

A higher removal efficiency of *Escherichia coli* during low-frequency ultrasound disintegration was obtained by Hawrylik (2019). She concluded that ultrasound has an effective impact on *E. coli*, in which a nearly 70% decrease in the amount of bacteria was obtained after 20 min of ultrasound at 40 kHz. Using acidification as a pretreatment method, Fukushi et al. (2003) found an effective reduction of *Salmonella* spp. in sewage sludge. Methods using chemical oxidants (advanced oxidation processes) have also positive effects in the elimination of microorganisms. After 2 h of reaction of persulfate (0.5 mM) activated by solar energy, a unit reduction of 6.0 log of bacteria in sewage sludge was registered (Ferreira et al. 2020). Luukkonen et al. (2020) used peracetic acid for the conditioning of municipal wastewater sludge. They concluded that with the increase in the dose of peracetic acid to 480 mg/l, a decrease in *Salmonella* sp. to an amount acceptable for use of such sludge as a fertilizer in Finland occurred. Moreover, using alkalization and ultrasound pretreatment methods on sludge, disintegration deactivations of *E. coli* and sulfite-reducing *Clostridia* were obtained (Martín-Díaz et al. 2017). Studies on the amount of energy input with microwaves (up to 7 kWh) into the sludge showed an approximately 5.90 log removal value of the analyzed pathogens (Mawioo et al. 2017).

For an organism to be considered an indicator, it should be characterized by common presence and survival in raw sludge. In addition, it must be easily cultured and easily identified. Therefore, it is difficult to choose one species of bacteria, which is the reason for using several indicator organisms. A potential indicator of the sanitary quality of sewage sludge, as mentioned in the literature (Sidhu and Toze 2009), is *Clostridium perfringens*. On the basis of microbiological tests, an elimination of *Clostridium perfringens* bacteria by each method of disintegration was observed. The number of *Clostridium perfringens* rods in WAS was reduced by 19.6% (after 30 min of disintegration by hydrodynamic cavitation), 7.8% (dry ice), and 38.4% (hybrid disintegration) (Figs. 2 and 3).

Similar effects were obtained for a decrease in the overall number of coliphages (Martín-Díaz et al. 2016). After 30 min of cavitation processes, the number was reduced by approximately 19.3%, while dry ice had a destructive effect of approximately 14.9% (Figs. 2 and 3). After the hybrid process, the number of coliphages changed from 5.77 log to 5.49 log cfu/g_d.w._ (Figs. 2 and 3).

The indicators of *Ascaris* sp.*, Trichuris* sp.*,* and *Toxocara* sp. are also being used in sanitary assessments of sewage sludge. Such indicators allow us to assess the agricultural usefulness of sewage sludge. Unfortunately, there is still a lack of sufficient information on their content in wastewater and sewage sludge and their survival during wastewater treatment processes.

Hydrodynamic disintegration and dry ice destroyed the eggs of helminths. The disintegration of WAS by hydrodynamic cavitation resulted in 26.4%, 22.8%, and 29.3% reductions in *Ascaris* sp., *Trichuris* sp., and *Toxocara* sp., respectively (Figs. 2 and 3). As a result of disintegration by dry ice, the overall egg number decreased. A decrease from 2.08 log to 2.03 log eggs/kg_d.w_ was observed for *Ascaris* sp., a decrease from 1.96 log to 1.88 log eggs/kg_d.w._ was observed for *Trichuris* sp. and a decrease from 3.05 log to 2.34 log eggs/kg_d.w._ was observed for *Toxocara* sp. (Figs. 2 and 3). A higher elimination effect was observed after the hybrid process was used.

In connection with the above, it can be clearly stated that the studied methods of disintegration contribute to the partial hygienization of sewage sludge. This is an important aspect because in activated sludge technology, the next stage (in most wastewater treatment plants—WWTPs) is the process of anaerobic stabilization.

The most commonly used process in WWTPs is stabilization under mesophilic conditions, which contributes to the decomposition and degradation of organic matter, biogas production and the diminution of pathogens. This process, unlike thermophilic digestion, has a low efficiency in the scope of sludge hygienization (Ruiz-Hernando et al. 2014; Carrere et al. 2016; Martín-Díaz et al. 2020). A low effectiveness of pathogenic bacterial inactivation in sewage sludge produced in Swedish treatment plants after digestion procedures (both thermophilic/mesophilic processes) was observed by Sahlström et al. (2004). Therefore, it is justified to look for solutions that contribute to the elimination of bacteria and parasite eggs before the fermentation process. One such possibility may be the use of disintegration methods. As we have shown in our earlier works (Grübel and Suschka 2015; Machnicka et al. 2015; Suschka and Grübel 2016; Grübel and Machnicka 2020; Li et al. 2021), the disintegration process effectively releases organic matter into the supernatant liquid of WAS, which contributes to the increased production of biogas. In connection with the above, we have undertaken research to determine the effectiveness of disintegration in removing selected bacterial indicators and parasite eggs.

According to the methodology (Fig. 1), the anaerobic stabilization process was performed on WAS with the addition of sludge after the hybrid disintegration process.

**Fig. 4:**
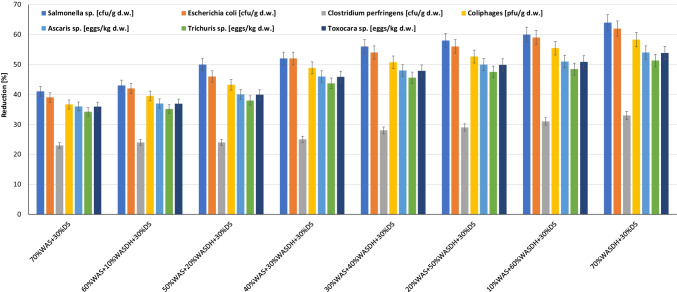
The impact of WAS disintegration and mesophilic digestion process on reduction of bacterial number and parasites eggs

Analyzing the above graph (Fig. 4), it can be noticed that with the increase in the volume of disintegrated WAS in the fermentation mixture, the reduction of the analyzed indicators increases. The reduction efficiency of *Salmonella* sp., *Escherichia coli*, *Clostridium perfringens*, and *coliphages* was better (from 1 to 23%) in mixtures with WASD compared to the control sample (70% WAS + 30% DS). Obviously, introducing only disintegrated sludge into the digestion chamber (70% WASD + 30% DS) is not economically and energetically justified from the technological point of view. Our previous research shows (Grübel and Suschka 2015) that the optimal dose of WASD is 30–40%, which definitely affects the production of biogas and biogas yield and is still economically justified.

Therefore, taking into account only the limited use of WASD in the fermentation mixture (30% vol.), the hygienization efficiency in relation to bacterial indicators was *Salmonella* sp. – 11%, *Escherichia coli* – 13%, *Clostridium perfringens* – 2%, and *coliphages* – 12%.

Similar effects were achieved with parasite eggs. Taking into account only 30% of the volume of WASD in the fermentation mixture, a reduction of 10% for *Ascaris* sp. and *Toxocara* sp. and 9.5% for *Trichuris* sp. in comparison to the blank sample (70% WAS + 30% DS) was attained.

Our investigations demonstrated an increase in the elimination of coliphages from 2.8 to 21.6% after the introduction of WASDH to fermentation processes. This result corresponds well to the results after mesophilic fermentation observed by Mandilara et al. (2006), which showed a reduction of 8.5%. Mocé-Llivina et al. (2003) claims that phages were much more resistant to thermal inactivation than bacterial indicators, with the exception of sulfite-reducing *Clostridia* spores. Somatic coliphages were significantly more resistant than *Salmonella choleraesuis*, *E. coli*, F-specific RNA phages, and enteroviruses. The *coliphages* survived markedly better than *Salmonella choleraesuis*, and the scope of inactivation indicated that naturally occurring bacteriophages can be used to monitor the deactivation of *E. coli* and *Salmonella* sp. Pino-Jelcic et al. (2006) observed the log inactivation of the fecal coliform of approximately 4.2 ± 0.4 for microwaved/digested sludge, whereas for conventionally heated/digested sludge and the control, the reductions were only 2.9 ± 0.5 and 1.5 ± 0.5, respectively. Less log deactivation was noted for *Salmonella* spp. in microwaved/digested sludge—approximately 2.0 ± 0.3, whereas for conventionally heated/digested sludge and the control, the reductions were 1.9 ± 0.2 and 1.1 ± 0.3, respectively. Similarly, Hong et al. (2006) concluded from bench-scale fermentation that the introduction of microwaved disintegrated sludge to an anaerobic digester was more efficient in the inactivation of fecal *coliforms* (average of ≥ 2.66 log reduction) in comparison to chambers fed with untreated sludge and externally heated sludge. They observed that Class A sludge can be produced only when the sludge before stabilization is heated using microwaves to 65 °C.

The research by Cella et al. (2016) showed an increase in fecal *coliform* removal from wastewater sludge depending on microwave intensification. In the fermenter chamber with the addition of microwaved sludge, 73.4% removal of *coliforms* was observed. Pike et al. (1988) described successful inactivation of *Salmonella duesseldorf* by mesophilic and thermophilic digestion but incomplete destruction of *Ascaris suum* ova. They concluded that the retention period influences the effectiveness of inactivation, and an increase in temperature from 36 to 48 °C increases the effectiveness by 3–4 times. Similar conclusions were obtained by Scheinemann et al. (2015). They found that 56 days of fermentation at 37 °C were required for a complete reduction of *Ascaris suum* ova. Moreover, Maya et al. (2010) noted that temperature plays an essential role in the survival of *Ascaris* eggs. Mesophilic processes are inefficient at totally eliminating viable nematode eggs (Venglovsky et al. 2006). Similarly, Wagner et al. (2008) and Orzi et al. (2015) found that the temperature and physico-chemical properties of the sludge in the fermenter influence the inhibition of microorganisms.

Very often, after pretreatment and anaerobic processes, there is a regrowth of bacterial indicators. According to Erkan and Sanin (2013) and Lepeuple et al. (2004), factors influencing the regrowth of bacteria are deficiency of nutrients, oxidation and osmotic stress and the presence of toxic substances. They are associated with the entry into the viable state of microorganisms, but they are not suitable for cultivation. This effect has not been found in bacteriophages. Martín-Díaz et al. (2017) concluded that alkali pretreatment and alkali and ultrasound pretreatments followed by anaerobic digestion repeatedly increased the number of *Clostridia*.

**Fig. 5:**
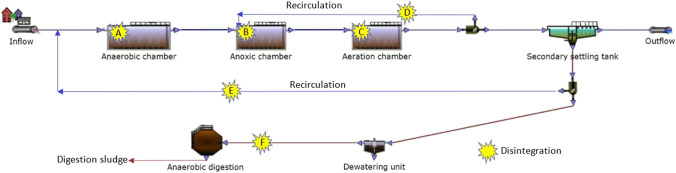
Potential places of application of disintegration method to the process of wastewater

The results presented confirm the effectiveness of destroying microorganisms and therefore WAS flocs. For example, in relation to a standard technological cycle of the wastewater purification process for complex biogenic and organic carbon elimination with the application of activated sludge, this process can be feasibility implemented in several places depending on the expected effects (Fig. 5).

Destruction by disintegration can be implemented in the process of internal (D) or external (E) recirculation together with the part sludge from the secondary settling tank to anaerobic zone (A), anoxic (B), and aerobic (C).

Introducing disintegrated WAS into the anaerobic chamber (A) may help increase phosphorus bacteria activity by increasing the charge of volatile fatty acids (VFA) in the chamber. In the anoxic zone (B) disintegrated WAS containing a high concentration of easily digestible carbon compound will assure to the denitrification bacteria the intensity of the nitrate reduction process in the situation of shortage of organic matter in wastewater flowing into the activated sludge reactor. The aerobic zone (C) through oxygenated metabolisms of heterotrophic microorganisms mainly decreases the concentration of non-settling, soluble and colloid carbon compounds in wastewater. The introduction of additional organic substrate will contribute to the rapid reproduction of bacteria, the increase of zoogleal clusters and the production of WAS flocks. On the other hand, applying WAS disintegration to the recirculation flow (D and E) can be used by additional/external carbon sources for metabolic processes.

Disintegrated WAS introduced with condensed surplus activated sludge (F) to digestion chamber could improve fermentation efficiency. According to the methodology, the impact of the hybrid method of disintegration on the mesophilic fermentation process (biogas production and methane concentration) was determined (Table 1).

**Table 1:** The impact of mixture composition on cumulative biogas production after mesophilic process

Parameter	Composition of digested mixture (%vol.)							
	70%WAS + 30%DS	60%WAS + 30%DS + 10%WASDH	50%WAS + 30%DS + 20%WASDH	40%WAS + 30%DS + 30%WASDH	30%WAS + 30%DS + 40%WASDH	20%WAS + 30%DS + 50%WASDH	10%WAS + 30%DS + 60%WASDH	30%DS + 70%WASDH
Cumulative biogas production (cm^3^/L)	2380 ± 78	2786 ± 83	3346 ± 103	3835 ± 124	3860 ± 132	3615 ± 115	3137 ± 92	2622 ± 82
CH_4_ (%)	61 ± 1	61 ± 2	62 ± 1	63 ± 2	64 ± 2	61 ± 1	62 ± 2	61 ± 1

*WAS* waste activated sludge*, DS* digested sludge (inoculum), *WASDH* sludge after hybrid disintegration; ± standard deviation

The obtained results confirmed that hybrid disintegration pretreatment (hydrodynamic cavitation and dry ice freezing/thawing) of only a part of WAS was substantiated. The volume of WASDH investigated was 10–70% in the digestion mixture. The highest effects of cumulative biogas production were obtained for 30% and 40% WASDH with increases of approximately 61.1% and 62.2%, respectively (Table 1).

The increasing amount of sewage sludge and the legislative regulation of its disposal have caused the need for developing new technologies to effectively process sewage sludge hygienization.

## Conclusions

Hybrid pretreatment processes (hydrodynamic disintegration and freezing/thawing) are a method to destroy bacteria and helminth eggs in waste activated sludge. Based on the microbiological and parasitological analyses, a significant reduction in pathogenic bacteria, coliphages and parasite eggs was observed. The number of *Salmonella* sp. in 1 g_d.w._ decreased by approximately 69.7%, that of *E. coli* decreased by 70.0%, that of *Clostridium perfringens* decreased by 38.4%, and that of *coliphages* decreased by 48.2% after hybrid disintegration. The disruption of WAS by hybrid pretreatment resulted in a reduction in the number of helminth eggs of *Ascaris* sp. (63.8%), *Trichuris* sp. (64.3%), and *Toxocara* sp. (66.4%).

The application of pretreatment methods and anaerobic digestion for WAS hygienization resulted in a higher reduction efficiency of *Salmonella* sp., *Escherichia coli*, *Clostridium perfringens*, and *coliphages*, from 1 to 23% in mixtures with WASD compared to the control sample (70% WAS + 30% DS). This indicates a lower transmission of sanitary indicators in the agricultural utilization of sludge after both pretreatments and digestion processes.

## Data Availability

Not applicable.
